# Socioeconomic status and sick leave granted for mental and somatic disorders: a prospective study of young adult twins 

**DOI:** 10.1186/s12889-015-1457-3

**Published:** 2015-02-12

**Authors:** Fartein Ask Torvik, Eivind Ystrom, Nikolai Czajkowski, Kristian Tambs, Espen Røysamb, Ragnhild Ørstavik, Gun Peggy Knudsen, Kenneth S Kendler, Ted Reichborn-Kjennerud

**Affiliations:** Division of Mental Health, Norwegian Institute of Public Health, Postbox 4404, Oslo, Nydalen 0403 Norway; Department of Psychology, University of Oslo, Postbox 1049 Blindern, Oslo, 0317 Norway; Department of Psychiatry, Virginia Commonwealth University, 1200 East Broad Street, P. O. Box 980710, Richmond, VA 23298-0710 USA; Virginia Institute for Psychiatric and Behavioral Genetics, Virginia Commonwealth University, Richmond, VA 23298-0126 USA; Institute of Clinical Medicine, University of Oslo, Postbox 1171 Blindern, Oslo, 0316 Norway

**Keywords:** Sick leave, Education, Income, Health inequalities, Twin study, Norway

## Abstract

**Background:**

Low socioeconomic status (SES), indicated by low income and education, has consistently been found to be a strong predictor of sick leave. Several possible pathways from SES to sick leave have been described in previous literature, but there are also evidence indicating that the association can be confounded by common underlying factors. This study utilizes a population-based sample of employed young adult twins to estimate (i) the degree to which education and income are prospectively related to sick leave granted for mental, somatic, and any disorder, and (ii) whether these associations are confounded by familial factors.

**Methods:**

Registry data on educational attainment and income at age 30 and subsequent sick leave were available for 6,103 employed young adult twins, among which there were 2,024 complete twin pairs. The average follow-up time was 6.57 years. Individual-level associations and fixed effects within twin pairs were estimated.

**Results:**

Low education and income were associated with sick leave granted for both mental and somatic disorders, and with sick leave granted for any disorder. Associations were attenuated within dizygotic twin pairs and reduced to non-significance within monozygotic twin pairs, suggesting influence of familial factors on the associations between SES and sick leave.

**Conclusions:**

Low SES is associated with a higher level of sick leave granted for both mental and somatic disorders among young adults, but these associations are confounded by factors that are common to co-twins. Education and income are therefore not likely to strongly affect sick leave in young adulthood.

## Background

Sick leave has high costs for employers and societies and is a putative cause of social exclusion [1,2]. Low socioeconomic status (SES), indicated by income, education, and occupation is a strong predictor of sick leave [3-8]. There is a well-documented social gradient in health [9,10], but SES differences in sick leave is not merely a byproduct of the health gradient, because differences in health across SES strata can only account for a minor part of SES differences in sick leave [7,11,12]. Several possible pathways from SES to sick leave have been described in previous literature. However, the results of some studies indicate that the association can be confounded, i.e., that other factors influence both SES and sick leave.

### How SES may affect sick leave

There are several mechanism by which SES may affect sick leave. Work attendance is influenced by both attendance ability (e.g. health) and motivation (e.g. job satisfaction, economic necessity) [13], and low SES may impair both. If low SES impairs health, it decreases the ability to attend work. Previous studies have found that low SES can affect physiological outcomes related to threat responses, such as systolic blood pressure [14,15]. In addition, low SES is associated with risky health behaviors [16] and harsher work environments. It is possible that SES affect these factors, and thereby ultimately sick leave. In line with this, work environment has been found to explain 20-60% of the SES differences in sick leave in the general population and samples of middle aged workers [4,6,8,17]. Harsh work conditions could also affect attendance ability because physically demanding jobs may not be adaptable to health impairments, while individuals with high SES jobs, typically in offices, are more often able to attend their jobs with similar impairments [18]. Low SES jobs are typically less rewarding, and this may influence attendance motivation and thereby sick leave. Such jobs often require high effort, and effort-reward imbalances have been found to predict sick leave [19,20]. Uncomfortable conditions could also affect attendance motivation, while high SES jobs are likely to elicit stronger internal motivation.

### Possible confounding of the association between SES and sick leave

Results from previous studies indicate that the association between SES and sick leave can be confounded. In observational studies, it is usually not possible to distinguish the effects of SES on sick leave from the effects of factors that influenced SES. Educational attainment, income and sick leave are all affected by genetic factors as well as environmental factors shared by family members [21-26]. It is thus possible that some of the same genetic and/or environmental familial factors underlie both SES and sick leave. Twin data can be useful in determining whether an association is likely to reflect a causal relationship, because it is possible to adjust for unobserved familial confounders [27]. By comparing sick leave among twins reared together, but discordant on SES, it is possible to observe the association between SES and sick leave adjusted for genetic and environmental factors associated with the family [27]. If the association between SES and sick leave is smaller within discordant twin pairs than it is in the population generally, confounding by familial factors is indicated. Associations remaining after adjusting for familial factors are therefore likely to lie closer to causal effects [28].

The SES differences in health are commonly explained by a combination of SES causing health differences [18,29,30] and selection into SES on factors related to health (“indirect selection”) [31,32]. If common factors, such as genetic or environmental familial factors, influence both health and SES, the association will be confounded. Previous studies have found the association between SES and several health measures to be confounded by familial factors [33,34]. Similarly, if factors influencing SES also affect sick leave, there will be spurious associations between SES and sick leave. Personality [35], self-control [36] and general ability [37] could represent background risk factors confounding the association between SES and sick leave. There can also be selection into jobs with hard working conditions. For example, one study found confounding by familial factors in the association between physical work characteristics and later disability pensioning granted for low back pain disorders [38], although such confounding was not seen in the relationship between psychosocial work characteristics and disability pensioning granted for low back pain [39] or mental [40] disorders. Importantly, one twin study observed the association between education, an important SES indicator, and disability pensioning, a phenomenon strongly related to sick leave, to be confounded by familial factors [41]. It is therefore possible that also the association between SES and sick leave is confounded.

### Aims

Educational attainment and income are related, but not interchangeable, indicators of SES [42]. This study utilizes a population sample of employed young adult twins and aims to (i) estimate the degree to which education and income are prospectively related to sick leave granted for mental and somatic disorders and sick leave granted for any disorder, and (ii) investigate whether these associations remain when adjusting for confounding genetic and environmental factors associated with the family, that is, whether the associations are consistent with a causal explanation.

## Methods

### Sample and design

The sample for the current study originated from the Norwegian Institute of Public Health Twin Panel (NIPHTP). The twins were identified through the national Medical Birth Registry, established January 1, 1967. The participants were twins born between 1967 and 1979 who took part in a large questionnaire study in 1998. All individuals were followed from approximately 30 years of age (details described below) and until the end of 2008, giving a mean follow-up time 6.57 years. By using national identification numbers issued to all Norwegians at birth, NIPHTP was linked to registries containing data on sick leave benefits, taxable income and highest educational attainment for the years 1998 to 2008.

Table 1 shows a summary of the construction of the sample and the length of follow-up by birth year. In brief, 12,700 twins were invited to the questionnaire study and 8,045 (63.3%) responded after one reminder. Of these, 7,710 were linked to sick leave data, while 335 twins withdrew from the study. In addition, 12 twins were excluded due to lack of data on zygosity, while 97 were excluded because they had emigrated. Of the remaining twins, 1,498 were not sufficiently employed at baseline and were excluded. Some of these were dead or already on disability pensioning, on long-term sick leave, or were still full-time students. Among the final sample of 6,103 twins (54.2% female), there were 2,024 complete pairs (384 monozygotic (MZ) male, 290 dizygotic (DZ) male, 453 MZ female, 332 DZ female, and 565 opposite sex twin pairs) and 2,055 single twins.

**Table 1:** **Construction of the sample and follow-up time by birth year**

	**Chronological time**												
**Birth year**	**‘98**	**‘99**	**‘00**	**‘01**	**‘02**	**‘03**	**‘04**	**‘05**	**‘06**	**‘07**	**‘08**	**N linked**	**N employed**
1967	+	+	+	+	+	+	+	+	+	+	+	697	565
1968	+	+	+	+	+	+	+	+	+	+	+	720	548
1969		+	+	+	+	+	+	+	+	+	+	726	580
1970			+	+	+	+	+	+	+	+	+	657	504
1971				+	+	+	+	+	+	+	+	639	512
1972					+	+	+	+	+	+	+	601	475
1973						+	+	+	+	+	+	622	481
1974							+	+	+	+	+	558	439
1975								+	+	+	+	541	423
1976									+	+	+	532	406
1977										+	+	448	350
1978											+	529	434
1979											+	440	350
**Total**	Mean follow up time = 6.57 years											7710	6103

The number of individuals linked to registry and the number of individuals sufficiently employed to be followed. Individuals are followed in the years indicated by ‘+’.

Zygosity classification was initially based on questionnaire data using discriminant analyses. Blood samples were obtained for a subsample of 676 like-sexed pairs, for whom zygosity was determined using 24 microsatellite markers. The results indicated a 97.5% correct original classification from questionnaire alone [43]. After correcting the originally misclassified pairs with DNA-based zygosity information, correct classification was estimated to be 98.0%.

#### Ethics

Consent was granted via the return of a completed questionnaire or specific consent form. The Regional Committees for Medical and Health Research Ethics approved of the study and the linkage with registry data from Statistics Norway.

### Measures

#### Education attainment

Data on the highest completed education were available annually from 1998 to 2008 from the Norwegian Educational Database administered by Statistics Norway. A large part of the sample was in their 20s during this period, which is the time most people complete their education and establish their workforce connection. We therefore decided to use educational attainment at age 30. In order to avoid excluding twins born in 1967 and 1979, for which educational data at age 30 were not available, educational attainment at age 31 (in 1998) and 29 (in 2008) was used. All individuals were followed from this age, rather than the date each individual became employed. This was done in order to avoid that people with low education should be systematically younger than those with high education. Also, this ensured that all co-twins were followed for the same length of time.

Educational attainment was registered with eight levels, ranging from no education to PhD or equivalent, according to the Norwegian Standard Classification of Education [44]. All included individuals had at least compulsory education, eliminating the lower end-point. PhDs (n = 31, 0.5%) were merged with master’s degrees, resulting in six educational levels.

#### Income

Annual information on income was registered in the Income Registry at Statistics Norway. Several definitions of income were available. For the current study we applied the variable “work related income”. As with educational attainment, we chose to use income the year the subjects turned 30 years old, or 29 or 31 if they were born in 1979 or 1967, respectively. Between 1998 and 2008 nominal incomes in Norway increased almost 60% [45]. Data on income were therefore adjusted in accordance with the average pay rise, to reflect 2008 equivalent income. Income was truncated at the 98th percentile.

#### Employment and sick leave

We obtained a longitudinal dataset, including detailed information on sick leave from 1998 to 2008 from The Historical-Event Database. Sick leaves exceeding 16 days are covered by the mandatory Norwegian Insurance Scheme for a duration of up to 52 weeks. Thus, the minimal sick leave period recorded in this study was 16 days. For each day from January 1, 1998 to December 31, 2008, we had information on whether each participant was registered as employed and at sick leave. Diagnosis set by physicians when sick leaves were granted were also available, coded according to the International Classification of Primary Care [46]. All individuals were followed from January 1 the year they turned 30 (or 29 or 31 if they were born in 1979 or 1967) and until the end of the observational period (December 31, 2008). This resulted in an average follow-up time of 6.57 years. Three sick leave variables were constructed: Sick leave granted for mental disorders (all diagnoses in the P-chapter), sick leave granted for somatic disorders (diagnoses in all other chapters), and sick leave granted for any disorder, which includes sick leaves with missing diagnosis. In order to account for the multiple sick leaves, sick leaves of different duration, and each individual’s level of employment, the variables for sick leave in each category were defined as a ratio (0–100%) of days lost to sick leave to the number of potential working days. Sick leave was only defined for individuals who had been employed and not at sick leave for at least 183 days during the first year they were followed. This criterion excluded individuals who already were on long lasting sick leave or rehabilitation, disabled individuals, full-time students, and other individuals with low labor-force participation.

### Statistical analyses

In accordance with the first aim, to estimate the associations between educational attainment, income and later sick leave, we ran linear regression analyses with random effects (Stata command *xtreg* option *re*), adjusting standard errors for dependency within pairs [27]. First, we estimated crude associations between sick leave and either education or income. Second, we adjusted these associations for sex and birth year. Third, we entered education, income and sick leave into the linear regression models simultaneously.

Our second aim was to investigate confounding by familial factors, that is, whether SES and sick leave were associated within twin pairs. We addressed this aim by estimating fixed effects (Stata command *xtreg* option *fe*) [47] within twin pairs, thereby adjusting for unobserved familial factors. MZ twins are genetically identical, whereas DZ twins share on average 50% of their genes out of those that vary in the population. In addition, twins who grow up together are matched on shared environmental factors, that is, environmental factors that make the twins more similar to each other, such as neighborhood, school, or dietary factors during pregnancy, but differ on non-shared environmental factors, that is, environmental factors that contribute to twin differences, such as lifestyle choices or individual experiences. For MZ twins, all effects of genes and shared environment are controlled for, while for DZ twins, effects of shared environment and 50% of genetic effects are controlled for. If there are no confounders in the environment unique to each twin, association within MZ twin pairs are estimates of causal effects [48,49]. Because an association is a prerequisite for causation, lack of association within MZ twin pairs is inconsistent with causal effects [48,49]. On the other hand, reduced associations would suggest confounding by familial factors, either genetic, environmental or both. If the associations are more strongly reduced among MZ twins pairs than among DZ twin pairs, confounding by genetic factors is indicated [28]. Only complete pairs were included in the within twin-pair analyses.

Interaction effects between sex and education, sex and income, birth year and education, and birth year and income were tested in analyses at the individual level and within twin pairs. All the analyses were repeated for each of the three sick leave categories: Sick leave granted for mental disorders, sick leave granted for somatic disorders, and sick leave granted for any disorder. The outcome variables were kept to their original scale, so that a 1.0 increase on a sick leave variable indicated a 1.0 percentage point increase in sick days to planned work days. Because small proportions of sick leave are more common than large, the sick leave variables were positively skewed. Bootstrapping with 1,000 repetitions was run to provide unbiased standard errors and confidence intervals. All analyses were also repeated with Poisson regression, and no differences of significance were found.

#### Software

The analyses were run in R 3.1.1 and Stata 12.1.

## Results

### Descriptive results

Table 2 shows the characteristics of the sample, as well as the proportion of workdays that were lost to sick leave granted for any disorder, somatic disorders, and mental disorders. Overall, 4.88% of workdays were lost to sick leave, of which 3.48% of workdays were lost to sick leave granted for somatic disorders and 0.93% to sick leave granted for mental disorders. The remaining sick leaves (0.47% of working days) had unknown diagnosis or were registered ambiguously in the registries. Sick leave was more common among women than among men, and more common among individuals with low education and income. For example, more than three times as many workdays were lost to sick leave among those in the lowest educational category compared to those in the highest.

**Table 2:** **Characteristics of the sample and proportion of workdays lost to sick leave (SL)**

		**SL any**		**SL somatic**		**SL mental**	
	**N**	**Mean**	**(SD)**	**Mean**	**(SD)**	**Mean**	**(SD)**
**Gender**							
Male	2794	2.35	(6.24)	1.67	(5.06)	0.51	(3.00)
Female	3309	7.01	(9.96)	5.02	(8.14)	1.28	(4.51)
**Year of birth (mean = 1972.43, SD = 3.71)**							
1967-1970	2233	5.11	(8.36)	3.37	(6.22)	1.09	(4.03)
1971-1975	2330	5.07	(8.57)	3.77	(7.12)	0.93	(3.76)
1976-1979	1540	4.25	(9.59)	3.22	(8.18)	0.70	(3.95)
**Educational attainment (mean = 3.96, SD = 1.40)**							
1 Lower secondary	180	8.46	(12.83)	5.18	(8.89)	2.23	(6.96)
2 Upper secondary, basic	748	7.34	(10.06)	5.24	(8.69)	1.61	(4.68)
3 Upper secondary, final year	1987	5.23	(9.21)	3.90	(7.54)	0.91	(4.00)
4 Post-secondary non-tertiary	220	3.77	(7.70)	2.88	(6.56)	0.58	(3.52)
5 Undergraduate studies	2171	4.41	(8.09)	3.08	(6.50)	0.84	(3.50)
6 Master’s degree or higher	797	2.46	(6.01)	1.69	(4.62)	0.37	(2.81)
**Income 2008 equivalent (mean = 376709 NOK, SD = 151151)**							
0 – 99,999 NOK	201	4.86	(10.37)	3.29	(8.53)	1.29	(5.38)
100,000 – 199,999 NOK	485	5.43	(9.20)	3.82	(7.87)	1.33	(4.41)
200,000 – 299,999 NOK	998	7.38	(10.60)	5.21	(8.35)	1.47	(5.15)
300,000 – 399,999 NOK	2026	5.69	(9.17)	4.14	(7.51)	1.01	(4.08)
400,000 – 499,999 NOK	1318	3.93	(7.86)	2.78	(6.38)	0.75	(3.34)
500,000 – 599,999 NOK	581	2.49	(5.56)	1.86	(4.71)	0.30	(1.77)
600,000 – 699,999 NOK	261	1.27	(3.69)	0.68	(1.81)	0.21	(1.25)
Above 700,000 NOK	233	1.38	(3.74)	1.07	(3.49)	0.16	(0.87)
**Total**	**6103**	**4.88**	**(8.77)**	**3.48**	**(7.10)**	**0.93**	**(3.91)**

SL granted for somatic disorders, mental disorders, and any disorder in percent. Average follow-up time is 6.57 years.

Note: The mean income corresponds to approximately €45000 or $60000; 100000 NOK (Norwegian kroner) equals approximately €12000 or $16000.

The mean difference between co-twins in educational attainment among the 2,024 complete twin pairs was 0.83 levels (SD = 0.97), which was higher among DZ twins (mean = 0.99, SD = 1.01) than among MZ twins (mean = 0.62, SD = 0.86). Among 837 MZ pairs, 498 had the same educational level, while 339 were discordant on education with on average 1.52 educational levels. Among 1187 DZ pairs, 485 were concordant on education, while 702 were discordant with an average of 1.67 educational levels. The mean difference in income within twin pairs was 135889 Norwegian kroner (NOK; 1 NOK ≈ €0.12 ≈ $0.16) (SD = 123,179, n = 2024), which was lower among MZs (mean = 109021, SD = 105990, n = 837) than DZs (mean = 154834, SD = 130743, n = 1,187). The correlation between income and educational attainment was 0.26 (95% C.I. 0.24 – 0.29, p <0.001) among all individuals. The mean difference in sick leave was 5.88 percentage points (SD = 8.82, n = 2024), and somewhat lower among MZ (mean = 5.09, SD = 7.97, n = 837) than DZ (mean = 6.44, SD = 9.34, n = 1187) twins.

### Regression analyses

Table 3 shows the estimates of the associations between educational attainment and income and sick leave. In the crude analyses (left column), income and education were associated with reduced sick leave granted for both mental and somatic disorders and a reduced sum of sick leave. Adjusted for sex and birth year (middle column), the associations between education and sick leave remained approximately the same, whereas the associations between income and sick leave became less pronounced. In the fully adjusted analyses (right column), where the effects of income and education are analyzed jointly, higher education was associated with reduced sick leave in all the categories, while higher income was only significantly associated with reduced sick leave granted for mental disorders. For each increment in educational level, the level of sick leave granted for somatic disorders decreased on average with 0.68 percentage points (b = −0.68, 95% CI −0.80, −0.55), sick leave granted for mental disorders decreased with 0.19 percentage points (b = −0.19, 95% CI −0.27, −0.10), and sick leave in total decreased with 0.88 percentage points (b = −0.88, 95% CI −1.04, −0.72).

**Table 3:** **Associations between education, income, and sick leave (SL)**

		**Crude**		**Adjusted** ^**a**^		**Fully adjusted** ^**b**^	
		**b**	**(95% C.I.)**	**b**	**(95% C.I.)**	**b**	**(95% C.I.)**
SL somatic	Education	**−0.67**	**(−0.79, −0.54)**	**−0.69**	**(−0.82, 0.57)**	**−0.68**	**(−0.80, −0.55)**
	Income	**−0.64**	**(−0.74, −0.53)**	**−0.24**	**(−0.35, −0.13)**	−0.05	(−0.15, 0.06)
SL mental	Education	**−0.23**	**(−0.31, −0.15)**	**−0.22**	**(−0.30, −0.14)**	**−0.19**	**(−0.27, −0.10)**
	Income	**−0.24**	**(−0.30, −0.18)**	**−0.17**	**(−0.24, −0.11)**	**−0.12**	**(−0.19, −0.05)**
SL any	Education	**−0.92**	**(−1.08, −0.76)**	**−0.92**	**(−1.08, −0.75)**	**−0.88**	**(−1.04, −0.72)**
	Income	**−0.91**	**(−1.04, −0.78)**	**−0.36**	**(−0.50, −0.23)**	−0.12	(−0.25, 0.02)
N		6103		6103		6103	

SL granted for somatic, mental, and any disorder. Increase or reduction in sick leave of percentage points per unit increase in education or income.

Notes: Bold values signify *p < 0.05*; ^a)^Adjusted for sex and birth year; ^b)^Income and education analyzed jointly, adjusted for sex and birth year.

Table 4 shows the within twin pair associations between sick leave and the predictors. Among DZ twins (left column), higher educational was associated with reduced sick leave granted for somatic disorders (b = −0.38, 95% CI −0.71, −0.05) and with reduced sick leave granted for any disorder (b = −0.56, 95% CI −0.94, −0.19). Education was not associated with sick leave granted for mental disorders (b = −0.06, 95% CI −0.25, 0.14). Income was not significantly associated with any of the sick leave variables within DZ twin pairs. Combining MZ and DZ pairs gives higher statistical power, but lower control for confounding familial factors than analyses of only MZ pairs. In these analyses (middle column), the only significant association was between higher education and reduced sick leave granted for any disorder (b = −0.35, 95% CI −0.68, −0.03). Within MZ twin pairs (right column), education (b = 0.19, 95% CI −0.37, 0.75) and income (b = 0.30, 95% CI −0.07, 0.66) was not significantly associated with sick leave granted for any disorder, or with the other sick leave variables. For sick leave granted for somatic or any disorder all estimates pointed in the opposite direction, that is, twins with higher SES tended to have more sick leave.

**Table 4:** **Within-twin pair associations between education, income, and sick leave (SL)**

		**Dizygotic pairs**		**All twin pairs**		**Monozygotic pairs**	
		**b**	**(95% C.I.)**	**b**	**(95% C.I.)**	**b**	**(95% C.I.)**
SL somatic	Education	**−0.38**	**(−0.71, −0.05)**	−0.21	(−0.48, 0.05)	0.21	(−0.21, 0.63)
	Income	0.05	(−0.22, 0.31)	0.09	(−0.11, 0.30)	0.19	(−0.10, 0.48)
SL mental	Education	−0.06	(−0.25, 0.14)	−0.08	(−0.24, 0.08)	−0.14	(−0.43, 0.15)
	Income	−0.09	(−0.25, 0.07)	−0.06	(−0.18, 0.05)	−0.02	(−0.18, 0.14)
SL any	Education	**−0.56**	**(−0.94, −0.19)**	**−0.35**	**(−0.68, −0.03)**	0.19	(−0.37, 0.75)
	Income	0.00	(−0.30, 0.30)	0.09	(−0.15, 0.33)	0.30	(−0.07, 0.66)
N pairs		1187		2024		837	

SL granted for somatic, mental, and any disorder. Increase or reduction of sick leave in percentage points per unit increase in education or income.

Notes: Bold values signify *p < 0.05*; Income and education were analyzed jointly, and results adjusted for sex and birth year.

**Figure 1:**
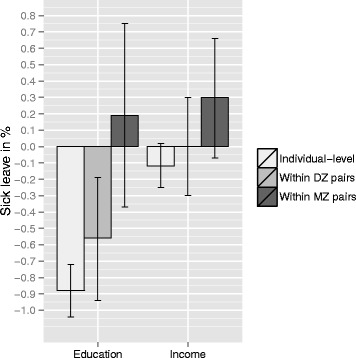
**Associations between education, income and sick leave before and after adjusting for familial factors.** Including 95% confidence intervals.

A graphical representation of the familial confounding is shown in Figure 1. The associations are reduced when moving from no correction for familial confounding, to partial correction in DZ pairs and complete correction for genetics and shared environment in MZ pairs. As can be seen, the effect of education within MZ twin pairs is clearly reduced compared to the individual-level analyses. The association with income was not significant in any of these analyses.

Interactions between sex and education, sex and income, birth year and education, and birth year and income were tested in all models. Out of 60 interaction tests, none were statistically significant at the α = 0.01 level.

## Discussion

The most important findings of the present study were that education and income were strongly and prospectively associated with sick leave among 30 year old persons in paid work, but that these associations appeared to be confounded by familial factors and therefore are probably not causal. We also found that low educational attainment was more predictive of sick leave than was low income in this age group.

The finding that low SES was associated with higher levels of sick leave replicates previous studies (e.g. [3,7]). We found SES differences in sick leave granted for both mental and somatic diagnoses, as well as with sick leave granted for any disorder. Education was more strongly related to sick leave than was income. A possible explanation is that education better reflects social status in young adulthood than income does, because returns to education are relatively low in Norway [50] and other egalitarian societies [47]. In addition, those with long education will have less work experience, which may be relevant for income in young adulthood. Nevertheless, income did have a significant association with sick leave granted for mental disorders independent of the effect of education.

Adjusting for factors shared by co-twins reduced the associations between SES and sick leave. Within MZ twin pairs, SES was not statistically significantly related to any of the sick leave variables. Therefore, the association between SES and sick leave is at least strongly, and possibly fully, confounded by familial factors. Several indices substantiate this: When the data were stratified on zygosity, the results in both the MZ sample and the DZ sample indicated familial confounding. The associations within DZ pairs, where the adjustment of genetic factors is partial, lay between the individual-level estimates and the associations within MZ pairs, where adjustment of familial factors is complete. In addition, for sick leave granted for any disorder and for sick leave granted for somatic disorders, the whole association with education disappeared in the within MZ pair analyses, and the estimates pointed non-significantly in the opposite direction, probably by chance. Non-significant findings cannot be taken as evidence that causal effects between SES and sick leave do not exist. However, considering the sizes of the confidence intervals, possible causal effects are clearly smaller than the crude associations between SES and sick leave.

As far as we are aware, no previous study has used twins to investigate whether the association between SES and sick leave is confounded or whether it is consistent with causality. Several causal mechanisms for the SES differences in sick leave have been described. Nevertheless, a few previous studies have found the associations between SES and constructs related to sick leave, such as health [33,34] or disability pensioning [41], to be confounded by familial factors. If it is true that low SES is not causally related to sick leave among young adults (in either direction), there must be a set of factors that influence both SES and sick leave. One may speculate that involved factors could be related to personality traits such as neuroticism or conscientiousness [51-53], general ability [37], or related to self-control [36]. The familial confounding is not likely to only consist of risk factors for specific disorders, because health differences do not explain all SES differences in sick leave [7].

If social class background rather than personally achieved education and income affected sick leave, one should expect to find similar results for MZ and DZ twins. This was not the case. The associations between sick leave and SES were stronger within DZs, who differ with respect to genetic factors, than within MZ pairs, who do not. This indicates that the familial factors confounding the associations between SES and sick leave are at least partly genetic.

The current findings concern education and income. Other indicators of SES, such as social capital or occupation, can have other types of relationships with sick leave. Some individuals hold jobs where they cannot fully utilize their education, whereas other individuals with low educational level achieve high-status positions. It is possible that twins who are discordant on education or income are more similar on occupational status than are unrelated individuals with similar difference in education and income.

Further, and importantly, if health differences between SES groups are due to lifestyle, diet, substance abuse, health literacy or wear and tear injuries, the effects of these are likely to accumulate over time. The effects of SES on sick leave may therefore be stronger among older individuals. Consistent with this line of reasoning, disability pensioning seems to be less heritable in older [54,55] compared to younger samples [24]. In addition, disability pensioning has been found to be associated with education within middle aged twin pairs [56].

### Limitations

This study has several strengths, such as prospective registry data on exposures and outcomes in a genetically informative population based sample of employed young adults. Nonetheless, some limitations must be mentioned. First, the power to detect statistically significant effects within MZ pairs was moderate. Although the association between education and sick leave was clearly reduced in this group, the lack of significance (in either direction) can be a type II error. Second, not all individuals have reached their highest education at age 30. However, only individuals who were working at baseline were included in the study, thus excluding for example full-time students, and individuals who had not entered work-life for other reasons, such as health. Third, co-twin control studies, like the present, cannot resolve issues of causality without reservations [48,49]. Nevertheless, they can adjust for unmeasured factors related to the family and thereby come a step closer to estimating causal effects than most other methods. Co-twin control studies are especially vulnerable to measurement error. The use of registry-based variables for both exposure and outcome was therefore especially important in this study. Fourth, in the current study we only considered long term sick leave (lasting for at least 16 days). Thus we do not know whether we would find exactly the same results for short-term sick leave. Finally, the results are best generalizable to Nordic countries and European countries with similar welfare schemes.

## Conclusions

Educational attainment, and, to a lesser extent, income, were associated with a prospective risk of sick leave granted for mental, somatic or any disorders among employed young adults. There were no associations within MZ twin pairs, indicating that confounding by factors that are common to co-twins appears to account for most of the SES differences in sick leave among young adults. Education and income are therefore not likely to strongly affect sick leave in young adulthood.

## Abbreviations

SES: Socioeconomic status
MZ: Monozygotic
DZ: Dizygotic
SL: Sick leave

## References

[1] OECD (2010). Sickness, Disability and Work: Breaking the Barriers.

[2] Bryngelson A (2009). Long-term sickness absence and social exclusion. Scand J Public Health.

[3] Allebeck P, Mastekaasa A (2004). Swedish Council on Technology Assessment in Health Care (SBU). Chapter 5. Risk factors for sick leave - general studies. Scand J Public Health.

[4] Melchior M, Krieger N, Kawachi I, Berkman LF, Niedhammer I, Goldberg M (2005). Work factors and occupational class disparities in sickness absence: findings from the GAZEL cohort study. Am J Public Health.

[5] Hansen H-T, Ingebrigtsen T (2008). Social class and sickness absence in Norway. Acta Sociol.

[6] Christensen KB, Labriola M, Lund T, Kivimäki M (2008). Explaining the social gradient in long-term sickness absence: a prospective study of Danish employees. J Epidemiol Community Health.

[7] Robroek SJW, van Lenthe FJ, Burdorf A (2013). The role of lifestyle, health, and work in educational inequalities in sick leave and productivity loss at work. Int Arch Occup Environ Health.

[8] Löve J, Hensing G, Holmgren K, Torén K (2013). Explaining the social gradient in sickness absence: a study of a general working population in Sweden. BMC Public Health.

[9] Marmot MG, Smith GD, Stansfeld S, Patel C, North F, Head J (1991). Health inequalities among British civil servants: the Whitehall II study. Lancet.

[10] Adler NE, Ostrove JM (1999). Socioeconomic status and health: what we know and what we don’t. Ann N Y Acad Sci.

[11] Østby KA, Ørstavik RE, Knudsen AK, Reichborn-Kjennerud T, Mykletun A (2011). Health problems account for a small part of the association between socioeconomic status and disability pension award. Results from the Hordaland Health Study. BMC Public Health.

[12] Kristensen TR, Jensen SM, Kreiner S, Mikkelsen S (2010). Socioeconomic status and duration and pattern of sickness absence. A 1-year follow-up study of 2331 hospital employees. BMC Public Health.

[13] Steers RM, Rhodes SR (1978). Major influences on employee attendance: a process model. J Appl Psychol.

[14] Kraus MW, Tan JJX, Tannenbaum MB (2013). The social ladder: a rank-based perspective on social class. Psychol Inq.

[15] Mendelson T, Thurston RC, Kubzansky LD (2008). Affective and cardiovascular effects of experimentally-induced social status. Health Psychol.

[16] Stringhini S, Sabia S, Shipley M, Brunner E, Nabi H, Kivimaki M (2010). Association of socioeconomic position with health behaviors and mortality. JAMA.

[17] Laaksonen M, Piha K, Rahkonen O, Martikainen P, Lahelma E (2010). Explaining occupational class differences in sickness absence: results from middle-aged municipal employees. J Epidemiol Community Health.

[18] Clougherty JE, Souza K, Cullen MR (2010). Work and its role in shaping the social gradient in health. Ann N Y Acad Sci.

[19] Peter R, Siegrist J (1997). Chronic work stress, sickness absence, and hypertension in middle managers: general or specific sociological explanations?. Soc Sci Med.

[20] Godin I, Kittel F (2004). Differential economic stability and psychosocial stress at work: associations with psychosomatic complaints and absenteeism. Soc Sci Med.

[21] Johnson W, Deary IJ, Silventoinen K, Tynelius P, Rasmussen F (2010). Family background buys an education in Minnesota but not in Sweden. Psychol Sci.

[22] Branigan AR, McCallum KJ, Freese J (2013). Variation in the heritability of educational attainment: an international meta-analysis. Soc Forces.

[23] Björklund A, Jäntti M, Solon G, Bowles S, Gintis H, Groves MO (2005). Chapter 4. Influences of Nature and Nurture on Earnings Variation: A Report on a Study of Various Sibling Types in Sweden. Unequal Chances Family Background and Econonomic Success.

[24] Gjerde LC, Knudsen GP, Czajkowski N, Gillespie N, Aggen SH, Røysamb E (2013). Genetic and environmental contributions to long-term sick leave and disability pension: a population-based study of young adult Norwegian twins. Twin Res Hum Genet.

[25] Svedberg P, Ropponen A, Alexanderson K, Lichtenstein P, Narusyte J (2012). Genetic susceptibility to sickness absence is similar among women and men: findings from a Swedish twin cohort. Twin Res Hum Genet.

[26] Bratberg E, Nilsen ØA, Vaage K (2013). Assessing the Intergenerational Correlation in Disability Pension Recipiency.

[27] Carlin JB, Gurrin LC, Sterne JA, Morley R, Dwyer T (2005). Regression models for twin studies: a critical review. Int J Epidemiol.

[28] McGue M, Osler M, Christensen K (2010). Causal inference and observational research: the utility of twins. Perspect Psychol Sci.

[29] Lindahl M (2002). Estimating the Effect of Income on Health and Mortality Using Lottery Prizes as Exogenous Source of Variation in Income.

[30] Leventhal T, Brooks-Gunn J (2000). The neighborhoods they live in: The effects of neighborhood residence on child and adolescent outcomes. Psychol Bull.

[31] Mackenbach JP (2005). Genetics and health inequalities: hypotheses and controversies. J Epidemiol Community Health.

[32] Goldman N (2001). Social inequalities in health disentangling the underlying mechanisms. Ann N Y Acad Sci.

[33] Fujiwara T, Kawachi I (2009). Is education causally related to better health? A twin fixed-effect study in the USA. Int J Epidemiol.

[34] Osler M, McGue M, Christensen K (2007). Socioeconomic position and twins’ health: a life-course analysis of 1266 pairs of middle-aged Danish twins. Int J Epidemiol.

[35] Roberts BW, Kuncel NR, Shiner R, Caspi A, Goldberg LR (2007). The power of personality: the comparative validity of personality traits, socioeconomic status, and cognitive ability for predicting important life outcomes. Perspect Psychol Sci.

[36] Moffitt TE, Arseneault L, Belsky D, Dickson N, Hancox RJ, Harrington H (2011). A gradient of childhood self-control predicts health, wealth, and public safety. Proc Natl Acad Sci U S A.

[37] Batty GD, Der G, Macintyre S, Deary IJ (2006). Does IQ explain socioeconomic inequalities in health? Evidence from a population based cohort study in the west of Scotland. BMJ.

[38] Ropponen A, Samuelsson Å, Alexanderson K, Svedberg P (2013). Register-based data of psychosocial working conditions and occupational groups as predictors of disability pension due to musculoskeletal diagnoses: a prospective cohort study of 24,543 Swedish twins. BMC Musculoskelet Disord.

[39] Ropponen A, Silventoinen K, Svedberg P, Alexanderson K, Huunan-Seppälä A, Koskenvuo K (2012). Effects of work and lifestyle on risk for future disability pension due to low back diagnoses: a 30-year prospective study of Finnish twins. J Occup Environ Med.

[40] Samuelsson A, Ropponen A, Alexanderson K, Svedberg P, Samuelsson Å (2013). Psychosocial working conditions, occupational groups, and risk of disability pension due to mental diagnoses: a cohort study of 43,000 Swedish twins. Scand J Work Environ Health.

[41] Samuelsson Å, Alexanderson K, Ropponen A, Lichtenstein P, Svedberg P (2012). Incidence of disability pension and associations with socio-demographic factors in a Swedish twin cohort. Soc Psychiatry Psychiatr Epidemiol.

[42] Martelin T (1994). Mortality by indicators of socioeconomic status among the Finnish elderly. Soc Sci Med.

[43] Harris JR, Magnus P, Tambs K (2006). The Norwegian institute of public health twin program of research: an update. Twin Res Hum Genet.

[44] Norway S (2003). Norwegian Standard Classification of Education Revised 2000.

[45] Table: 05220: Average pay for all employees, full-time equivalents, by work hours, educational level and sex [Tabell: 05220: Gjennomsnittlig månedslønn for ansatte, heltidsekvivalenter, etter arbeidstid, utdanningsnivå og kjønn (avslutta serie)] [https://www.ssb.no/statistikkbanken/selectvarval/Define.asp?subjectcode=&ProductId=&MainTable=LonnArbUtdann&nvl=&PLanguage=0&nyTmpVar=true&CMSSubjectArea=arbeid-og-lonn&KortNavnWeb=lonnansatt&StatVariant=&checked=true]

[46] WONCA (2005). ICPC-2-R: International Classification of Primary Care.

[47] Miller P, Mulvey C, Martin N, Findings US, Miller BP (2010). What do twins studies reveal about the economic returns to education? A comparison of australian and U.S. findings. Am Econ Rev.

[48] Frisell T, Öberg S, Kuja-Halkola R, Sjölander A (2012). Sibling comparison designs: bias from non-shared confounders and measurement error. Epidemiology.

[49] Sjölander A, Frisell T, Öberg S (2012). Causal interpretation of between-within models for twin research. Epidemiol Method.

[50] Bhuller M, Mogstad M, Salvanes KG (2011). Life-Cycle Bias and the Returns to Schooling in Current and Lifetime Earnings. Discussion Paper No. 666.

[51] Conte JM, Jacobs RR (2009). Validity evidence linking polychronicity and big five personality dimensions to absence, lateness, and supervisory performance ratings. Hum Perform.

[52] Esteban BL, Ortuño MMS, Izquierdo MG, Hernández JAR, Maldonado AL (2012). Personality traits and sick leave in workers diagnosed with nonorganic neck pain. Span J Psychol.

[53] Ones DS, Viswesvaran C, Schmidt FL (2003). Personality and absenteeism: a meta-analysis of integrity tests. Eur J Pers.

[54] Harkonmäki K, Silventoinen K, Levälahti E, Pitkäniemi J, Huunan-Seppälä A, Klaukka T (2008). The genetic liability to disability retirement: a 30-year follow-up study of 24,000 Finnish twins. PLoS One.

[55] Narusyte J, Ropponen A, Silventoinen K, Alexanderson K, Kaprio J, Samuelsson Å (2011). Genetic liability to disability pension in women and men: a prospective population-based twin study. PLoS One.

[56] Samuelsson Å, Ropponen A, Alexanderson K, Lichtenstein P, Svedberg P (2012). Disability pension among Swedish twins–prevalence over 16 years and associations with sociodemographic factors in 1992. J Occup Environ Med.

